# Supplementary material for: Mieap forms membrane-less organelles involved in cardiolipin metabolism
Source: iScience. 2024 Jan 17;27(2):108916. doi: 10.1016/j.isci.2024.108916 (PMC10845071; doi:10.1016/j.isci.2024.108916)
Supplement: Document S1. Figures S1–S16 [file mmc1.pdf]

iScience, Volume *27*

Supplemental information

Mieap forms membrane-less organelles involved in cardiolipin metabolism

Naoki Ikari, Katsuko Honjo, Yoko Sagami, Yasuyuki Nakamura, and Hirofumi Arakawa

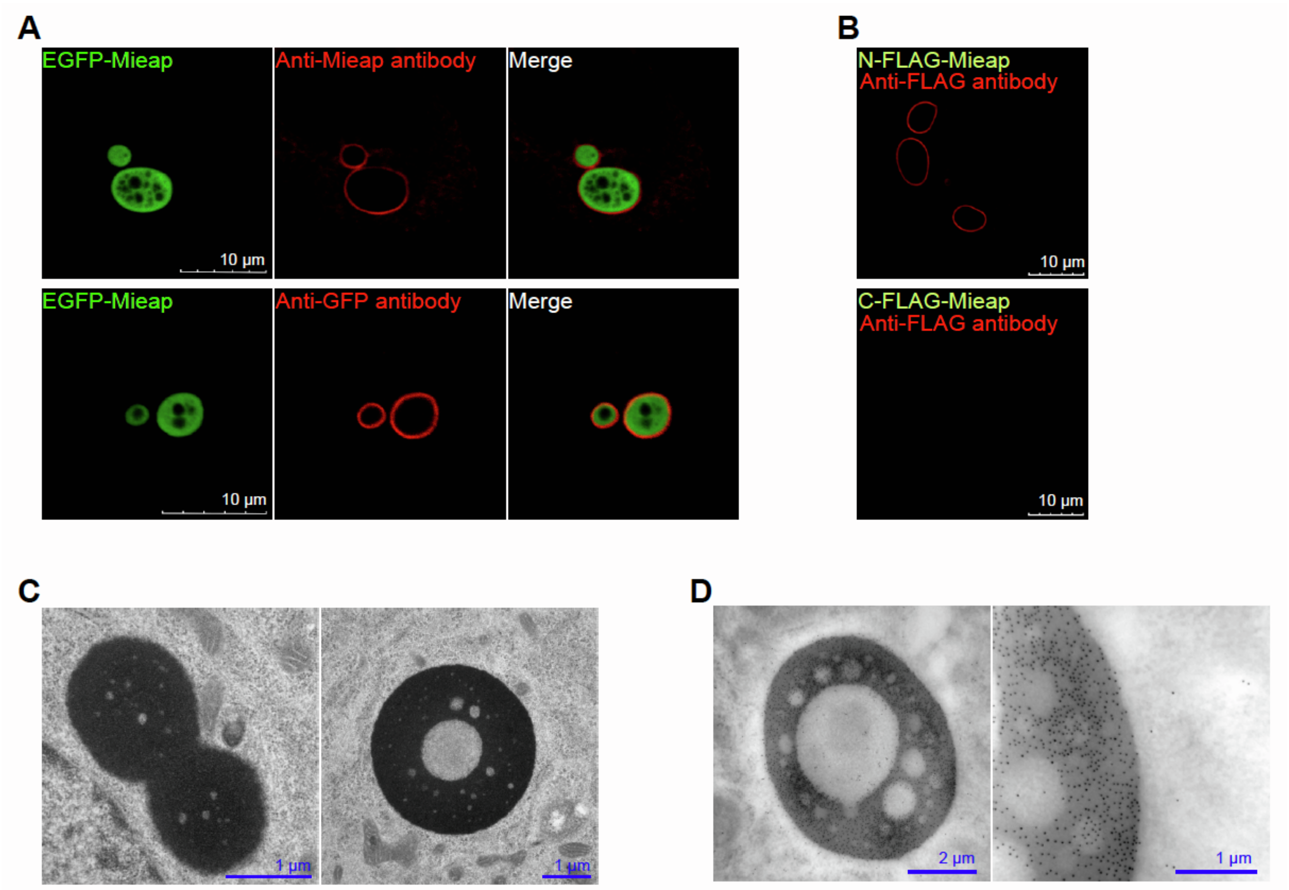

Figure S1. Mieap forms biomolecular condensates, related to Figure 1

(A) Comparative imaging of EGFP-Mieap condensates, visualized with both EGFP-Mieap and immunofluorescence (IF) using anti-Mieap antibody (upper panel) or anti-GFP antibody (lower panel) in A549 cells. Scale bars, 10 μm.

(B) IF imaging of N-FLAG-Mieap condensates (upper panel) or C-FLAG-Mieap condentates (lower panel) using anti-FLAG antibody in A549 cells. Scale bars, 10 μm.

(C) Transmission electron microscopy of Mieap condensates stained with osmium (OsO_4). Scale bars, 1 μm.

(D) Post-embedding immunoelectron microscopy of Mieap condensates using anti-Mieap antibody. Scale bars, 2 μm (left panel) and 1 μm (right panel).

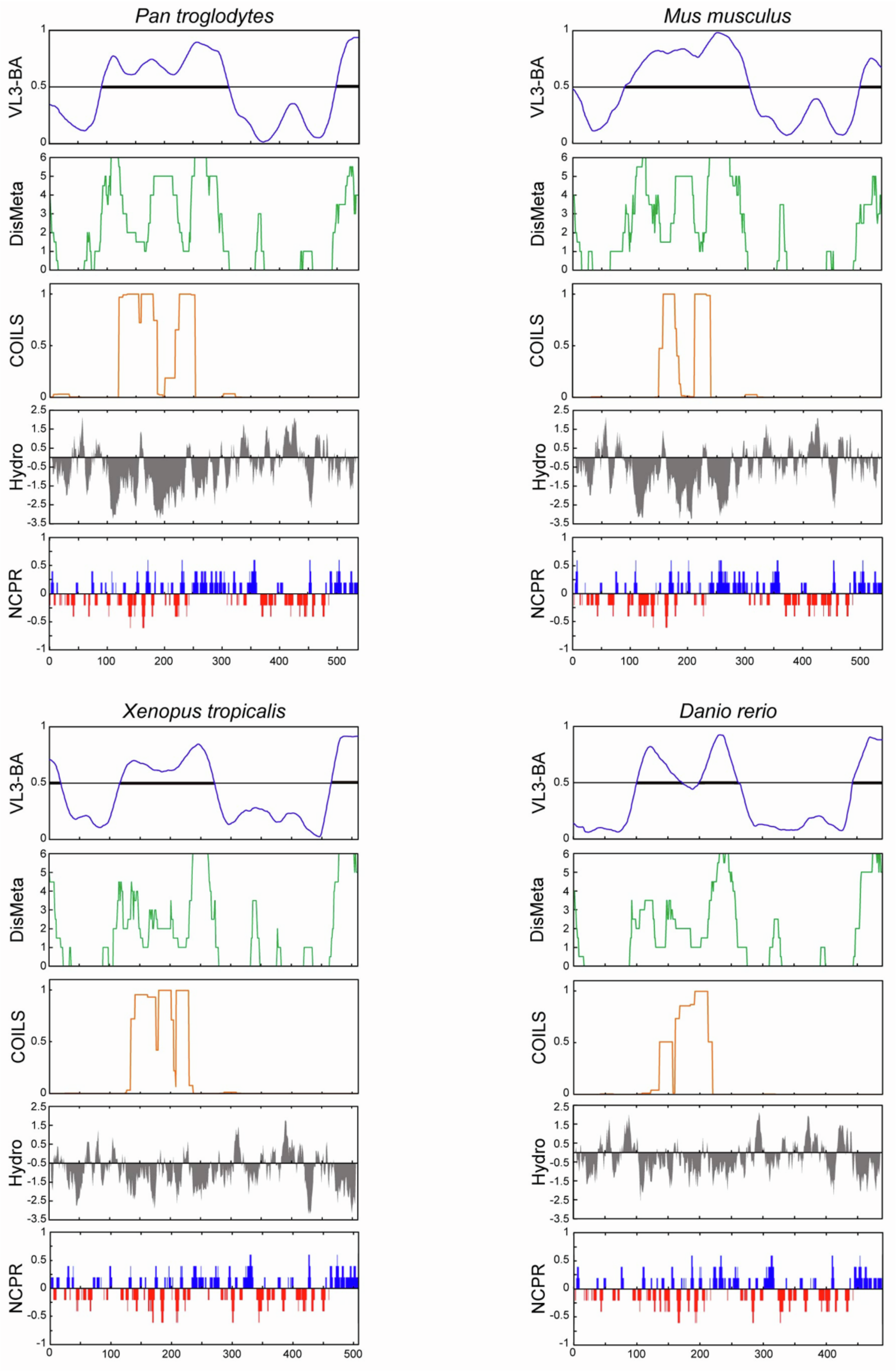

Figure S2. Analyses of Mieap orthologs, related to Figure 2

Amino acid sequence analyses of representative Mieap orthologs, as in Figure 2E.

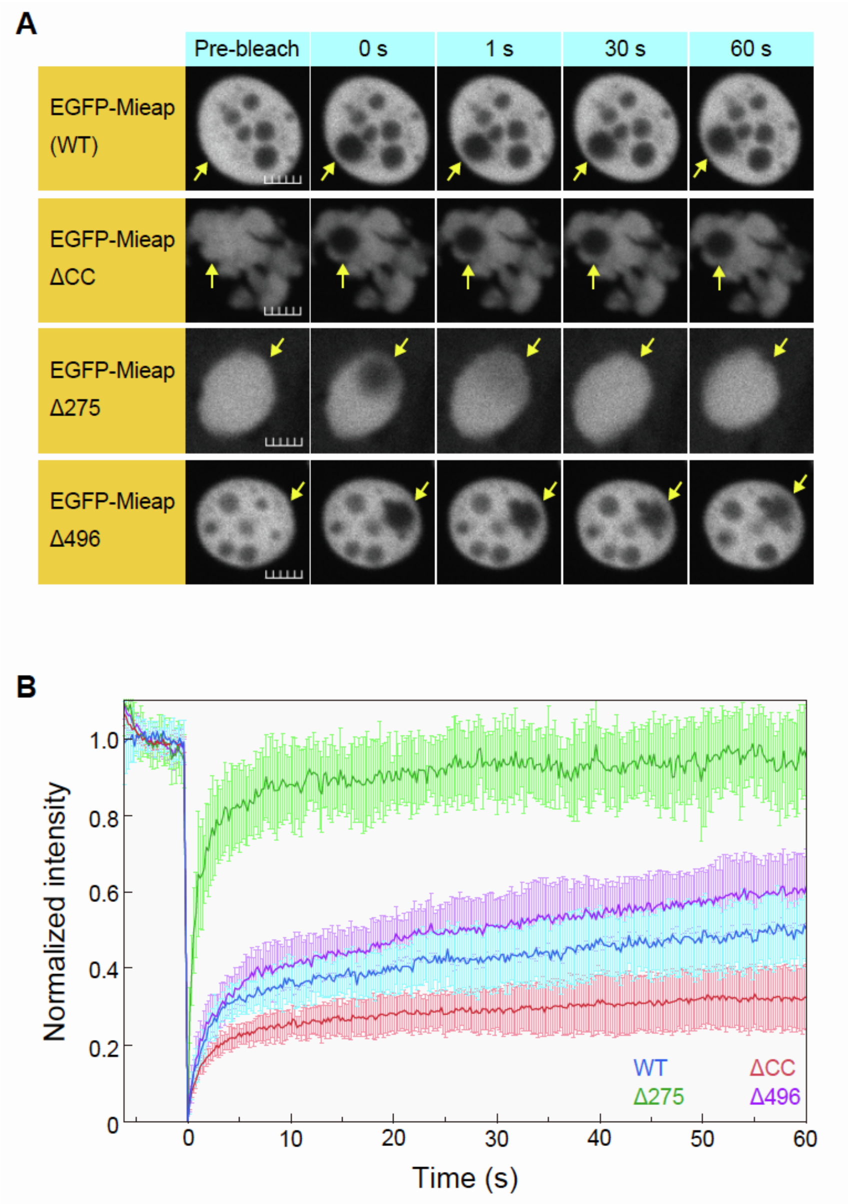

Figure S3. FRAP analysis of condensates formed by EGFP-Mieap and three deletion mutants, related to Figure 3

(A) Representative FRAP images of condensates formed by EGFP-Mieap (WT) and three deletion mutants (ΔCC, Δ275, and Δ496) in A549 cells. Each condensate was subjected to spot-bleaching using a 488-nm laser at 10% laser power with an 11.6 μs/μm exposure time and followed up for 60 s. Bleached areas are indicated by yellow arrows. Scale bars, 2 μm.

(B) Plotting of normalized average fluorescence recovery in the FRAP experiment with weaker laser exposure. Laser power was weakened to 1.4% and the exposure time was shortened to 1.4 μs/μm. n = 15 condensates for each construct. Data shown are means ±SD.

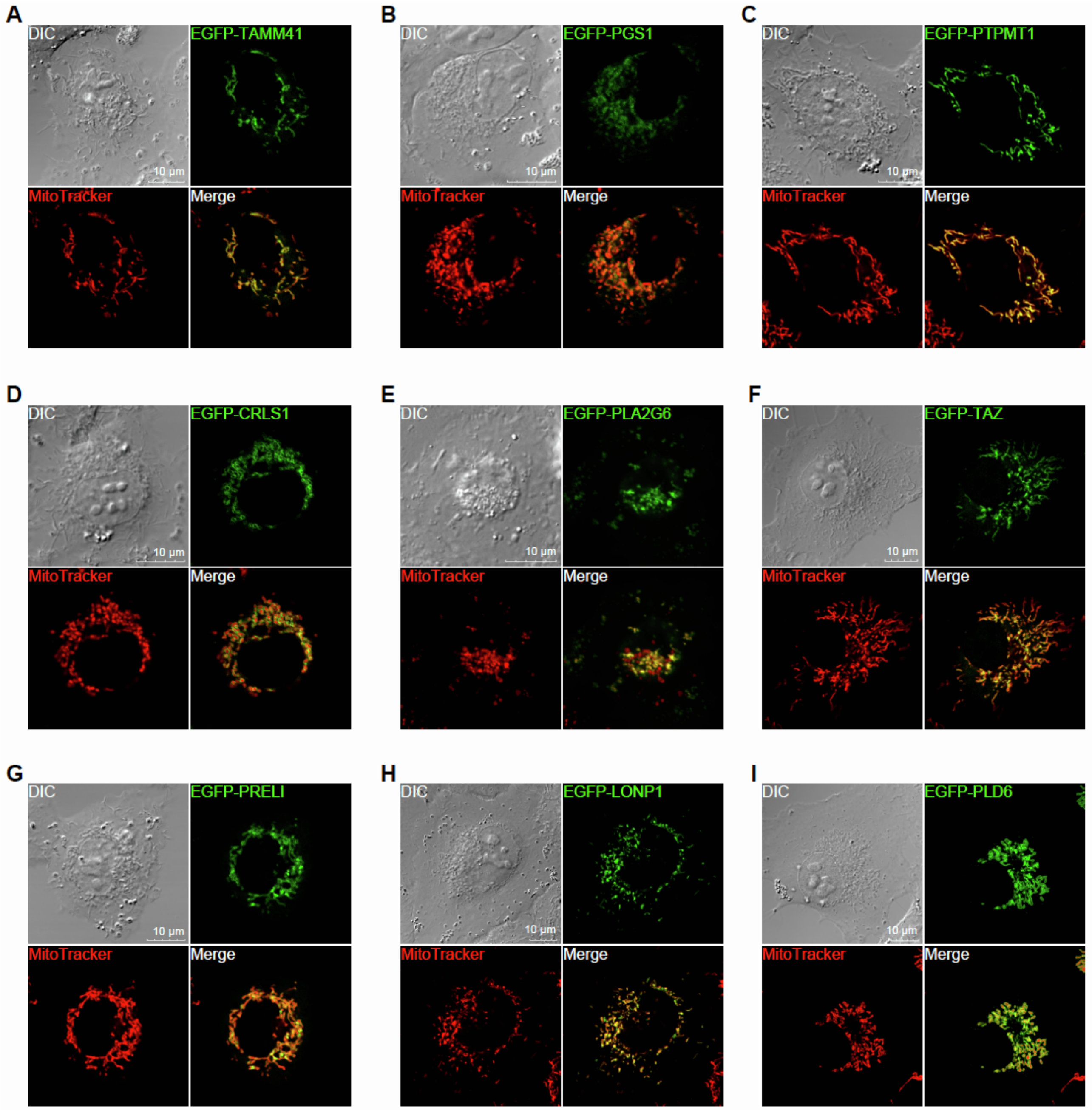

Figure S4. Verification of subcellular localization of fluorescently labeled proteins, related to Figure 4

(A – I) Subcellular localization of EGFP-TAMM41 (A), EGFP-PGS1 (B), EGFP-PTPMT1 (C), EGFP-CRLS1 (D), EGFP-PLA2G6 (E), EGFP-TAZ (F), EGFP-PRELI (G), EGFP-LONP1(H), and EGFP-PLD6 (I) verified by confocal live cell imaging, compared with localization of MitoTracker Red in A549 cells. Scale bars, 10 μm.

Mieap: Droplet driver

P_{LLPS} = 0.8481 (P_{LLPS} = or > 0.60)

Droplet-promoting region: 91-137, 147-299

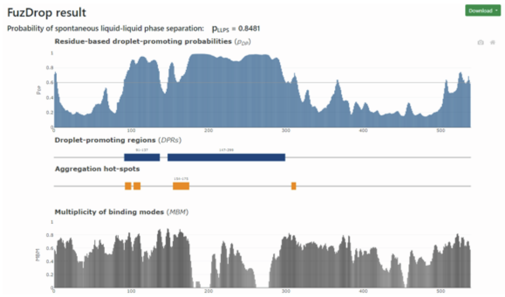

PGS1: Droplet client

P_{LLPS} = 0.1729 (P_{LLPS} < 0.60)

Droplet-promoting region: 32-43, 333-346 (P_{DP} = or > 0.60 for at least 10 consecutive residues)

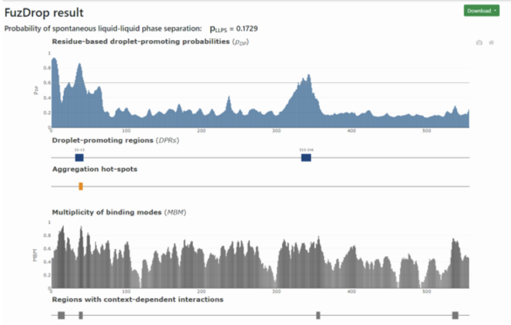

CRLS1: Droplet client

P_{LLPS} = 0.2964 (P_{LLPS} < 0.60)

Droplet-promoting region: 53-104 (P_{DP} = or > 0.60 for at least 10 consecutive residues)

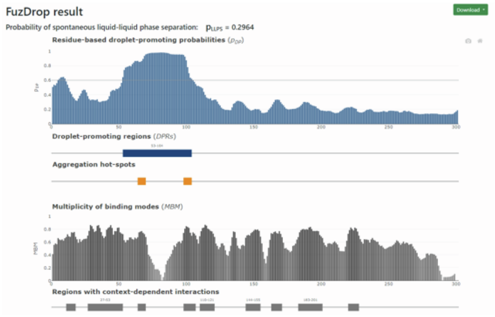

PLA2G6: Droplet client

P_{LLPS} = 0.3761 (P_{LLPS} < 0.60)

Droplet-promoting region: 276-288, 337-351, 417-445 (P_{DP} = or > 0.60 for at least 10 consecutive residues)

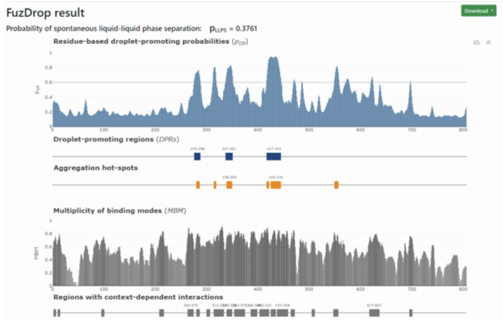

TAZ: Droplet client

P_{LLPS} = 0.2078 (P_{LLPS} < 0.60)

Droplet-promoting region: 131-155, 281-292 (P_{DP} = or > 0.60 for at least 10 consecutive residues)

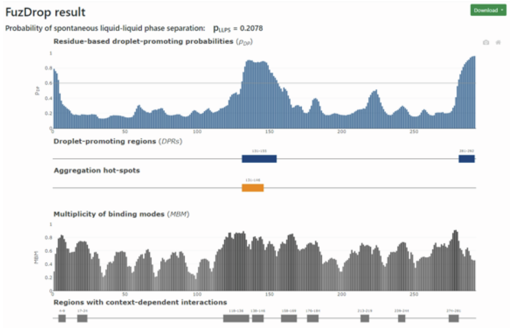

PRELI: Droplet client

P_{LLPS} = 0.1476 (P_{LLPS} < 0.60)

Droplet-promoting region: 190-219 (P_{DP} = or > 0.60 for at least 10 consecutive residues)

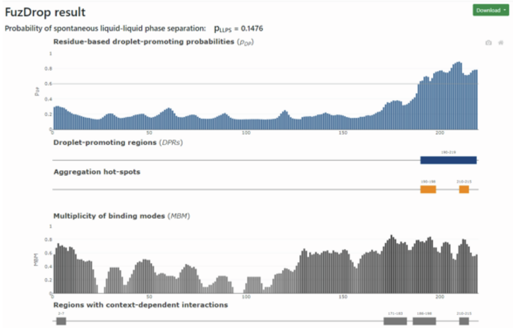

Figure S5. The FuzDrop program predicts that Mieap acts as a driver of the MLOs, and that PRELI, PGS1, CRLS1, PLA2G6, and TAZ act as clients of the MLOs, related to Figure 4

Utilizing a protein sequence, the FuzDrop method (Nucleic Acids Research. 50: w337-w344, 2022.) predicts the probability of spontaneous liquid-liquid phase separation of proteins, and also provides a sequence-based score to identify the regions that promote this process. The results are shown as the probability of spontaneous liquid-liquid phase separation (P_{LLPS}), which informs on the ability of protein to drive droplet formation. According to the program of the FuzDrop method, proteins with P_{LLPS} = or >0.60 likely spontaneously phase separate and serve as droplet drivers. The results are also shown as the droplet-promoting probabilities of residues (P_{DP}), the values of which vary between 0 and 1 and inform on the ability of residues to be involved in droplet-interactions. On the basis of these parameter and threshold, the program predicts droplet-driver and droplet-client proteins as shown below.

Droplet driver: P_{LLPS} = or > 0.60

Droplet client: P_{LLPS} < 0.60 and P_{DP} = or > 0.60 for at least 10 consecutive residues

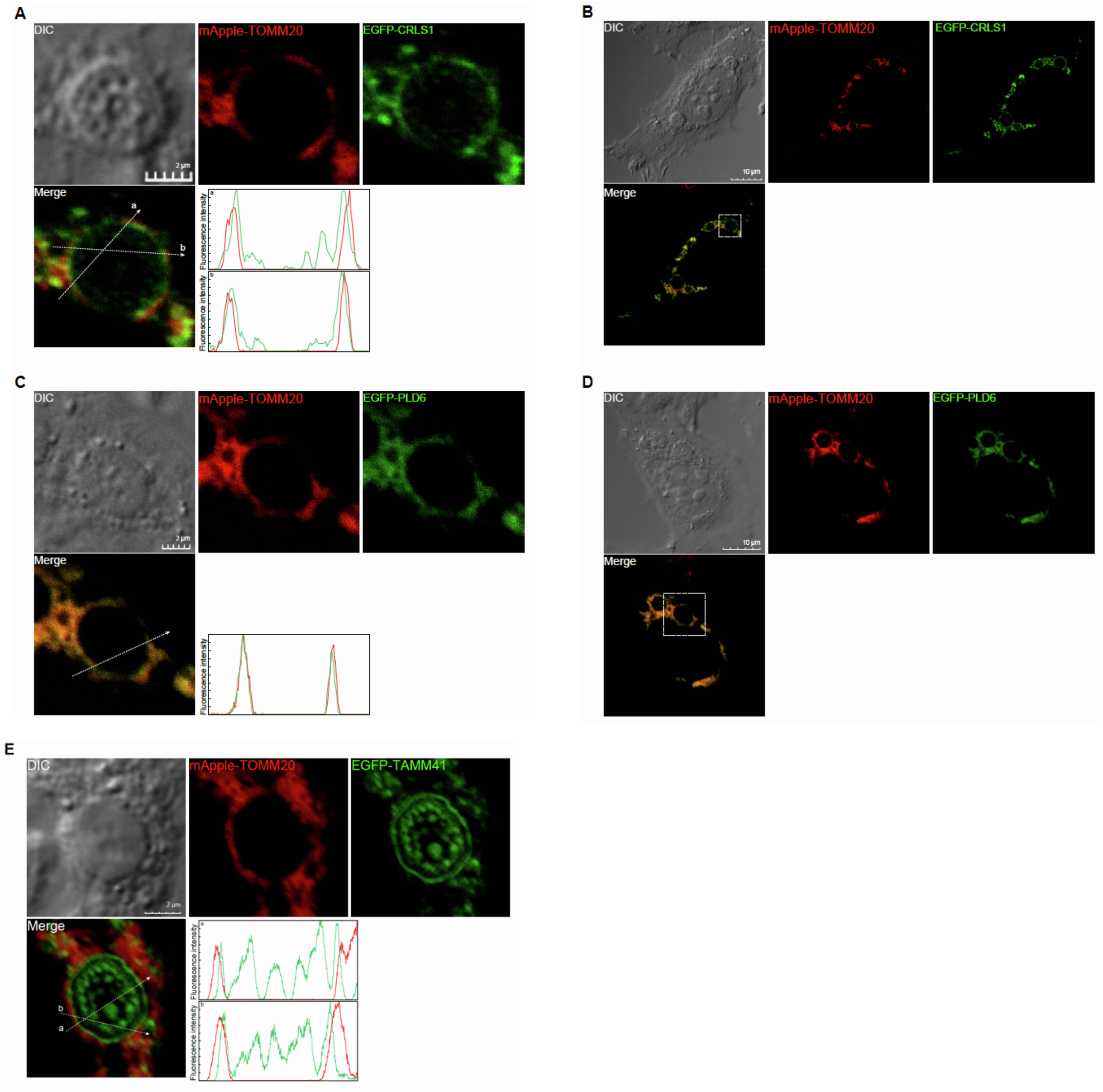

Figure S6. Mi-BCs are surrounded by both mitochondrial outer and inner membrane proteins, related to Figure 5

(A, B) Live-cell imaging showing the spatial relationship between Mi-BCs and the mitochondrial outer and inner membranes labeled simultaneously with mApple-TOMM20 (outer mitochondrial membrane) and EGFP-CRLS1 (inner mitochondrial membrane). (A) Lower right: line-scans of fluorescence intensities along the dashed arrows. (C, D) Live-cell imaging showing the spatial relationship between Mi-BCs and the mitochondrial outer membranes labeled simultaneously with mApple-TOMM20 and EGFP-PLD6 (outer mitochondrial membrane). (C) Lower right: line-scan of fluorescence intensities along the dashed arrow. (E) Live-cell imaging showing the spatial relationship between Mi-BCs and the mitochondrial outer and inner membranes labeled simultaneously with mApple-TOMM20 and EGFP-TAMM41 (inner mitochondrial membrane). Lower right: line-scans of fluorescence intensities along the dashed arrows. Scale bars, 2 μm (A, C, E) and 10 μm (B, D).

LS174T (related to Figure 7A and 7B)

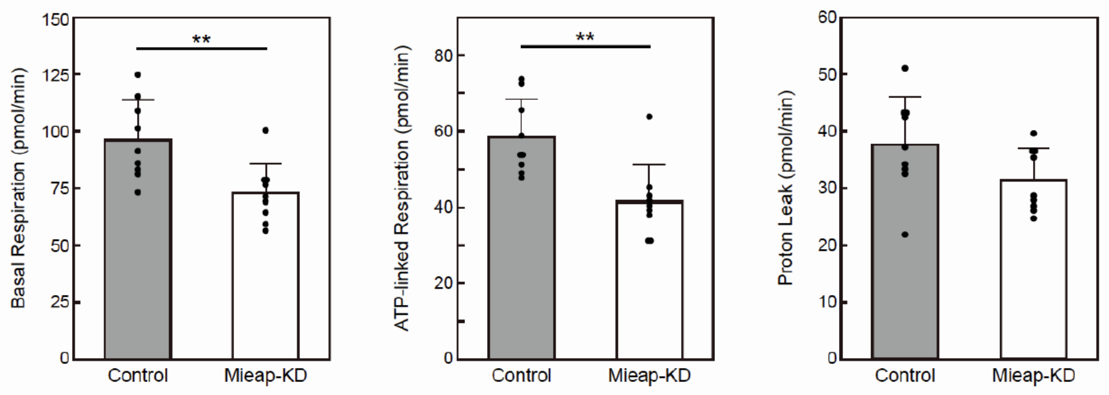

HCT116 (related to Figure 7G – 7J)

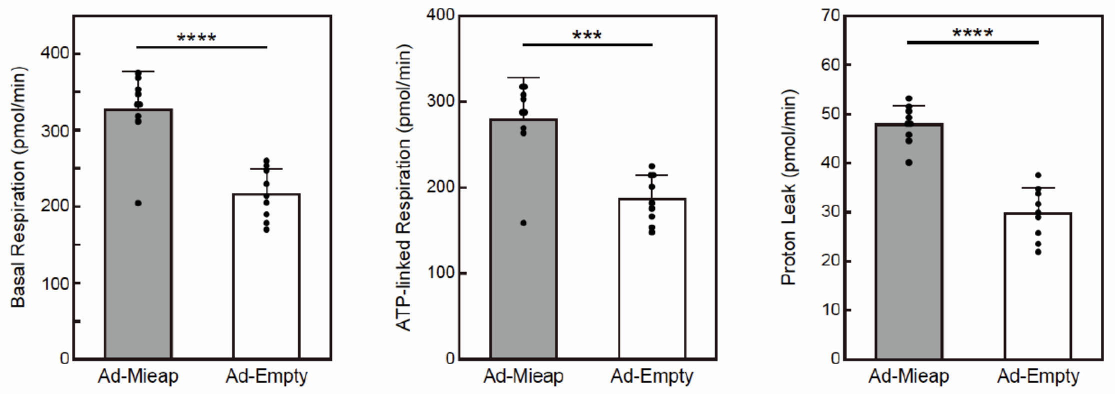

Figure S7. Quantitative assessment of basal respiration, ATP-linked respiration, and proton leak OCR, related to Figure 7

Quantitative assessment of basic OCR (left), ATP-linked OCR (middle), and proton leak OCR (right) of the LS174T cells (upper) and the HCT116 cells (lower). Data are shown as means ± SD (n = 9). * $p < 0.05$, ** $p < 0.01$, *** $p < 0.001$, **** $p < 0.0001$, two tailed Student's t-test.

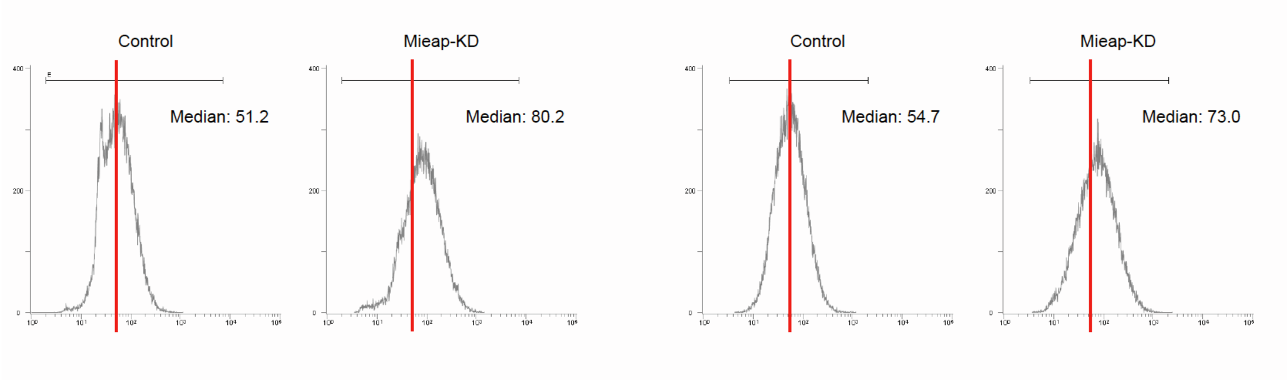

Figure S8. Reactive oxygen species (ROS) levels increase in Mieap-KD cells, related to Figure 7

ROS levels of LS174T-cont and Mieap-KD cells cultured under normal conditions, analyzed by flow cytometry using 2′,7′-dichlorofluorescein-diacetate. Data from duplicate experiments are shown.

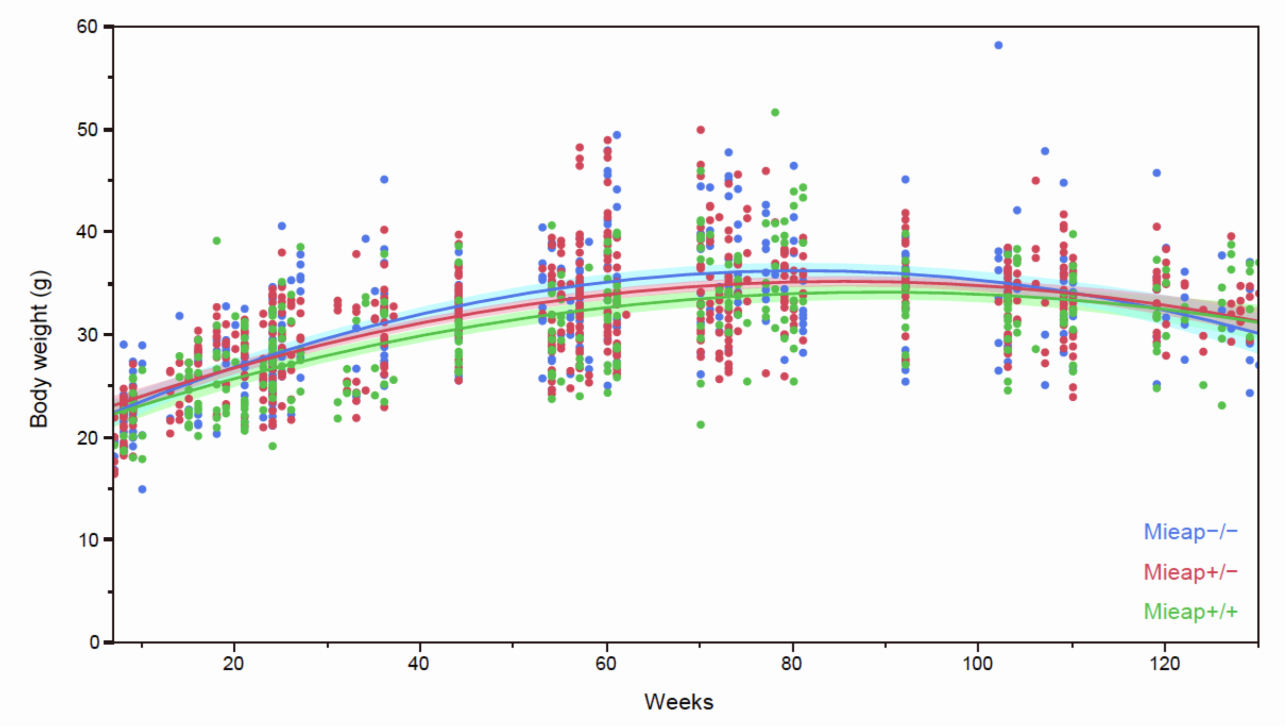

Figure S9. Mieap prevents obesity, related to Figure 8

Body weights of 1,225 Mieap$^{+/+}$, Mieap$^{+/-}$, and Mieap$^{-/-}$ mice (n = 315 Mieap$^{+/+}$, 571 Mieap$^{+/-}$, and 339 Mieap$^{-/-}$ mice, 7-130 weeks of age) were weighed. Dots and quadratic regression curves with 95% confidence intervals are shown for each genotype.

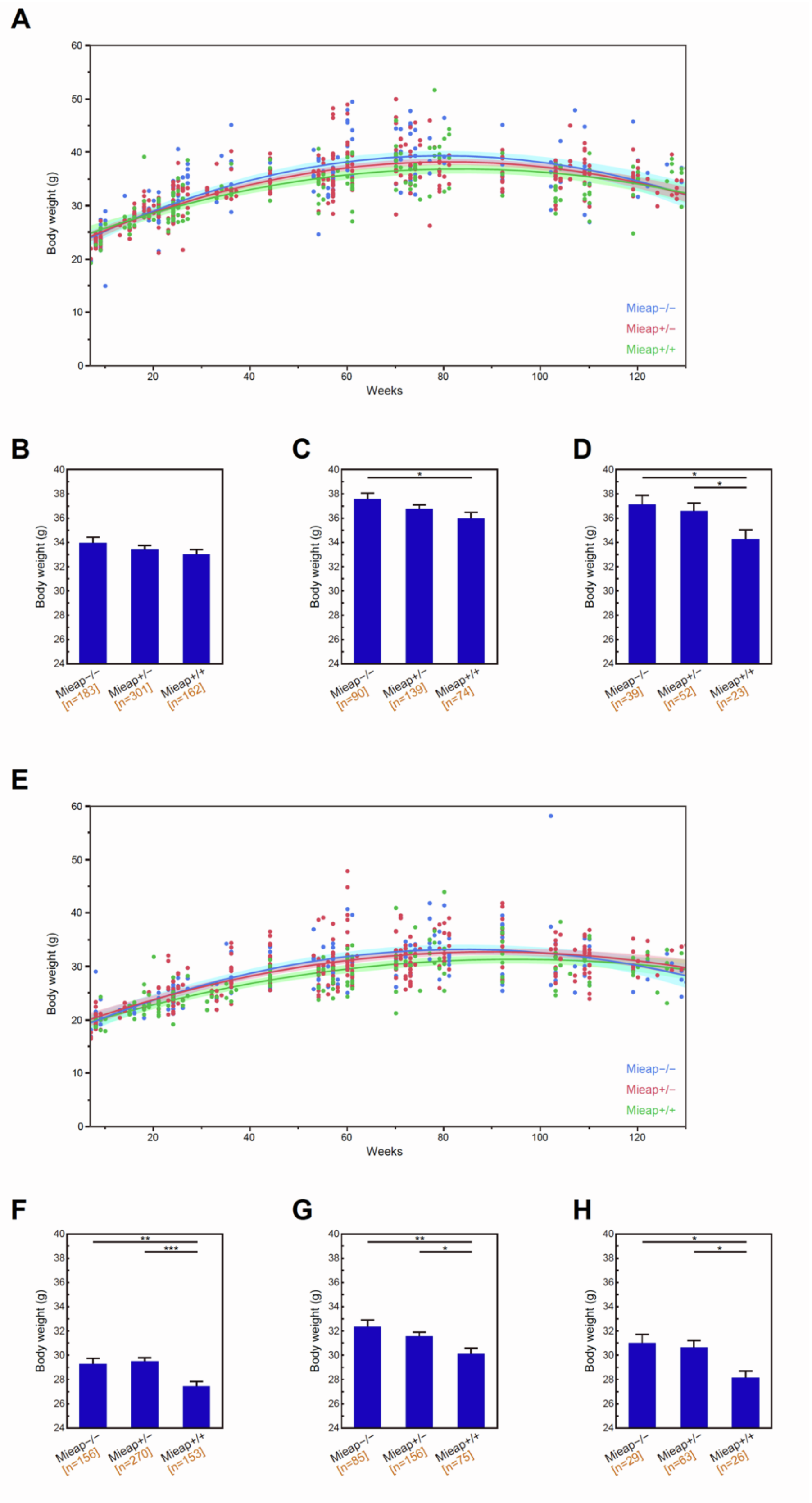
A
B
C
D
E
F
G
H
Body weight (g)
Weeks
Mieap−/−
Mieap+/−
Mieap+/+
[n=183]
[n=301]
[n=162]
[n=90]
[n=139]
[n=74]
[n=39]
[n=52]
[n=23]
[n=156]
[n=270]
[n=153]
[n=85]
[n=156]
[n=75]
[n=29]
[n=63]
[n=26]

Figure S10. Subgroup analysis by gender of the association between Mieap-deficiency and obesity, related to Figure 8

(A) Body weights of 646 $Mieap^{+/+}$, $Mieap^{+/-}$, and $Mieap^{-/-}$ male mice (n = 162 $Mieap^{+/+}$, 301 $Mieap^{+/-}$, and 183 $Mieap^{-/-}$ mice, 7-130 weeks of age). Dots and quadratic regression curves with 95% confidence intervals are shown for each genotype.

(B) Body weights of $Mieap^{+/+}$, $Mieap^{+/-}$, and $Mieap^{-/-}$ male mice (7-130 weeks of age) in (A).

(C) Body weights of $Mieap^{+/+}$, $Mieap^{+/-}$, and $Mieap^{-/-}$ male mice from middle-age and older mice (44-104 weeks of age) in (A).

(D) Body weights of $Mieap^{+/+}$, $Mieap^{+/-}$, and $Mieap^{-/-}$ male mice around 60 (53-62) weeks of age in (A).

(E) Body weights of 579 $Mieap^{+/+}$, $Mieap^{+/-}$, and $Mieap^{-/-}$ female mice (n = 153 $Mieap^{+/+}$, 270 $Mieap^{+/-}$, and 156 $Mieap^{-/-}$ mice, 7-130 weeks of age). Dots and quadratic regression curves with 95% confidence intervals are shown for each genotype.

(F) Body weights of $Mieap^{+/+}$, $Mieap^{+/-}$, and $Mieap^{-/-}$ female mice (7-130 weeks of age) in (E).

(G) Body weights of $Mieap^{+/+}$, $Mieap^{+/-}$, and $Mieap^{-/-}$ female mice from middle-age and older mice (44-104 weeks of age) in (E).

(H) Body weights of $Mieap^{+/+}$, $Mieap^{+/-}$, and $Mieap^{-/-}$ female mice around 60 (53-62) weeks of age in (E).

(B – D, and F – H) Data shown are means ±SE. *$p < 0.05$, **$p < 0.01$, ***$p < 0.001$, two tailed Student's t-test.

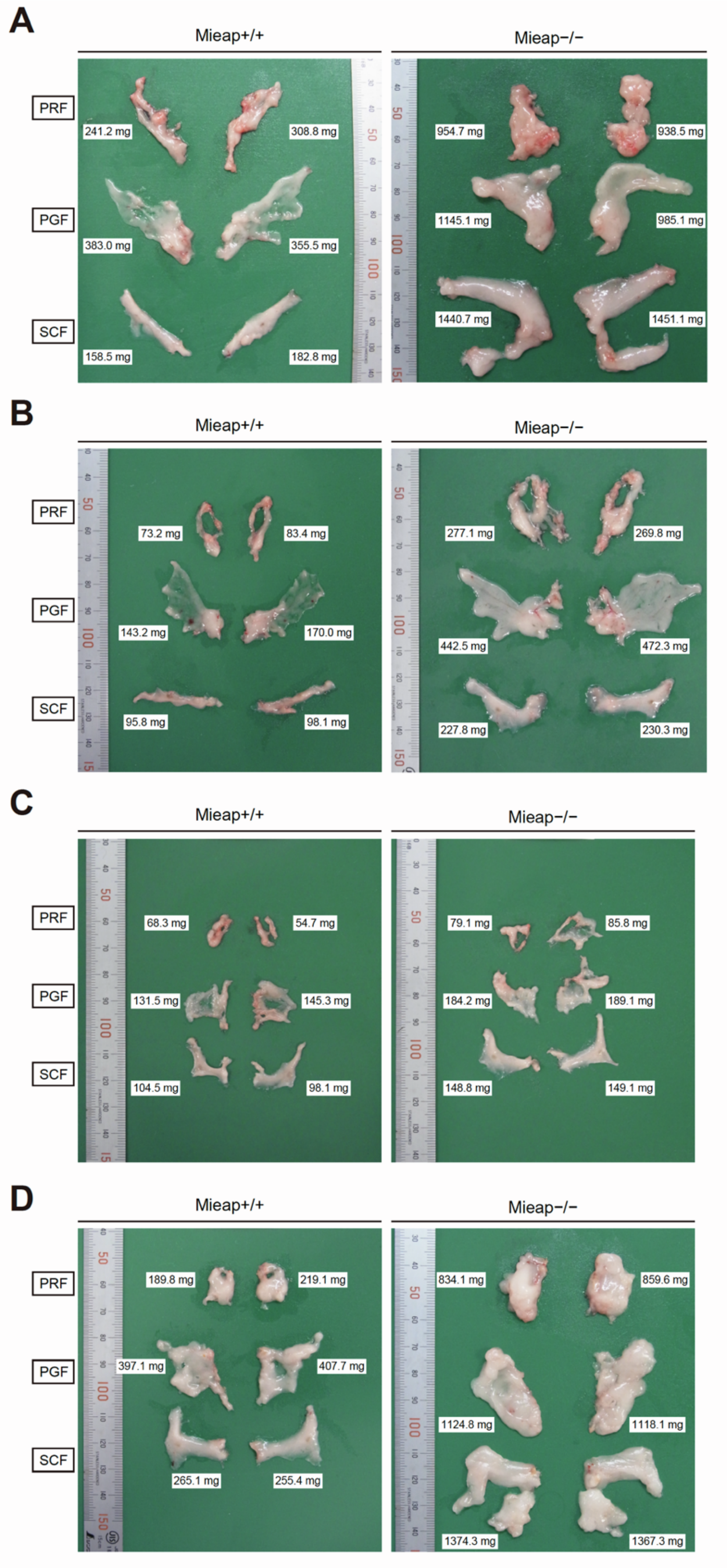
A
Mieap+/+
Mieap−/−
PRF
PGF
SCF
241.2 mg
308.8 mg
383.0 mg
355.5 mg
158.5 mg
182.8 mg
954.7 mg
938.5 mg
1145.1 mg
985.1 mg
1440.7 mg
1451.1 mg
B
Mieap+/+
Mieap−/−
PRF
PGF
SCF
73.2 mg
83.4 mg
143.2 mg
170.0 mg
95.8 mg
98.1 mg
277.1 mg
269.8 mg
442.5 mg
472.3 mg
227.8 mg
230.3 mg
C
Mieap+/+
Mieap−/−
PRF
PGF
SCF
68.3 mg
54.7 mg
131.5 mg
145.3 mg
104.5 mg
98.1 mg
79.1 mg
85.8 mg
184.2 mg
189.1 mg
148.8 mg
149.1 mg
D
Mieap+/+
Mieap−/−
PRF
PGF
SCF
189.8 mg
219.1 mg
397.1 mg
407.7 mg
265.1 mg
255.4 mg
834.1 mg
859.6 mg
1124.8 mg
1118.1 mg
1374.3 mg
1367.3 mg

Figure S11. Comparison of fat composition between Mieap$^{+/+}$ and Mieap$^{-/-}$ mice, related to Figure 8

(A – D) Amount of subcutaneous fat (SCF) and intra-abdominal fat (perigonadal fat and perirenal fat) (PGF and PRF) in the representative four litter pairs (Mieap$^{+/+}$ and Mieap$^{-/-}$) are shown.

(A) 73-week-old male mice, Mieap$^{+/+}$ (+/+) vs. Mieap$^{-/-}$ (−/−); Body Weight (BW): 35.59g (+/+) vs. 55.33g (−/−), SCF: 158.5mg (Left: L) and 182.8mg (Right: R) (+/+) vs.1440.7mg (L) and 1451.1mg (R) (−/−), PGF: 383.0mg (L) and 355.3mg (R) (+/+) vs. 1145.1mg (left) and 985.1mg (R) (−/−), PRF: 241.2mg (L) and 308.8mg (R) (+/+) vs. 954.7mg (L) and 938.5mg (R) (−/−).

(B) 90-week-old male mice, BW: 32.54g (+/+) vs. 39.03g (−/−), SCF: 95.8mg (L) and 98.1mg (R) (+/+) vs. 227.8mg (L) and 230.3mg (R) (−/−), PGF: 143.2mg (L) and 170.0mg (R) (+/+) vs. 442.5mg (L) and 472.3mg (R) (−/−), PRF: 73.2mg (L) and 83.4mg (R) (+/+) vs. 277.1mg (L) and 269.8mg (R) (−/−).

(C) 22-week-old female mice, BW: 20.78g (+/+) vs. 24.21g (−/−), SCF: 104.5mg (L) and 98.1mg (R) (+/+) vs. 148.8mg (L) and 149.1mg (R) (−/−), PGF: 131.5mg (L) and 145.3mg (R) (+/+) vs. 184.2mg (L) and 189.1mg (R) (−/−), PRF: 68.3mg (L) and 54.7mg (R) (+/+) vs. 79.1mg (L) and 85.8mg (R) (−/−).

(D) 81-week-old female mice, BW: 26.40g (+/+) vs. 39.31g (−/−), SCF: 265.1mg (L) and 255.4mg (R) (+/+) vs. 1374.3mg (L) and 1367.3mg (R) (−/−), PGF: 397.1mg (L) and 407.7mg (R) (+/+) vs. 1124.8mg (L) and 1118.1mg (R) (−/−), PRF: 189.8mg (L) and 219.1mg (R) (+/+) vs. 834.1mg (L) and 859.6mg (R) (−/−).

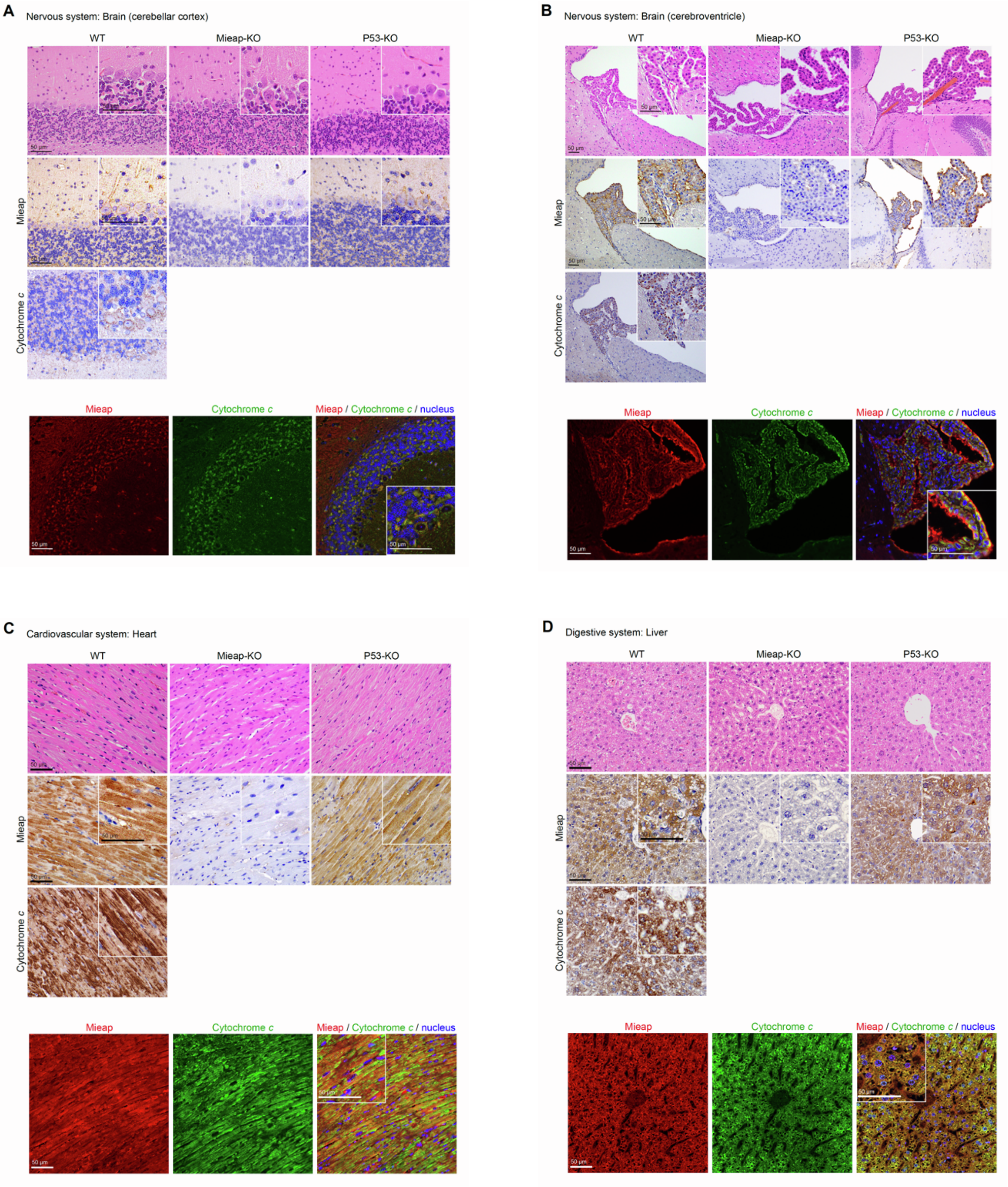

Figure S12. Specific expression of endogenous Mieap protein in brain (cerebellar cortex), brain (cerebroventricle), heart, and liver evaluated by histological analysis, related to Figure 8.

(A – D) Hematoxylin and eosin staining (upper panels), diaminobenzidine-based immunohistochemistry (IHC) (middle panels), and immunofluorescence (IF) (lower panels) for Mieap and cytochrome c performed for the WT, Mieap-KO, and p53-KO mice tissues/organs: (A) brain (cerebellar cortex), (B) brain (cerebroventricle), (C) heart, and (D) liver. Scale bars, 50 μm.

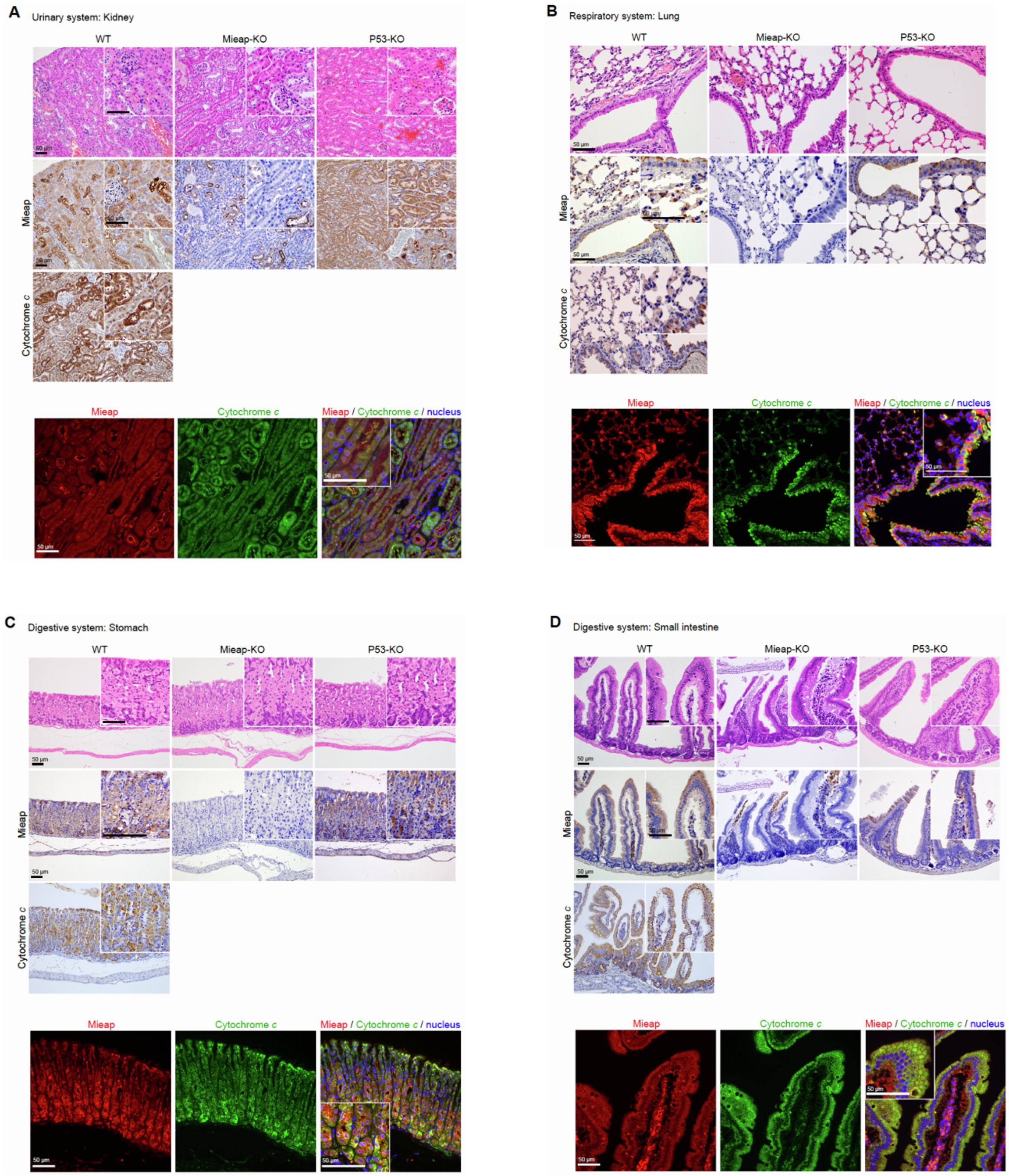

Figure S13. Specific expression of endogenous Mieap protein in kidney, lung, stomach, and small intestine evaluated by histological analysis, related to Figure 8.

(A – D) Hematoxylin and eosin staining (upper panels), diaminobenzidine-based immunohistochemistry (IHC) (middle panels), and immunofluorescence (IF) (lower panels) for Mieap and cytochrome c performed for the WT, Mieap-KO, and p53-KO mice tissues/organs: (A) kidney, (B) lung, (C) stomach, and (D) small intestine. Scale bars, 50 μm.

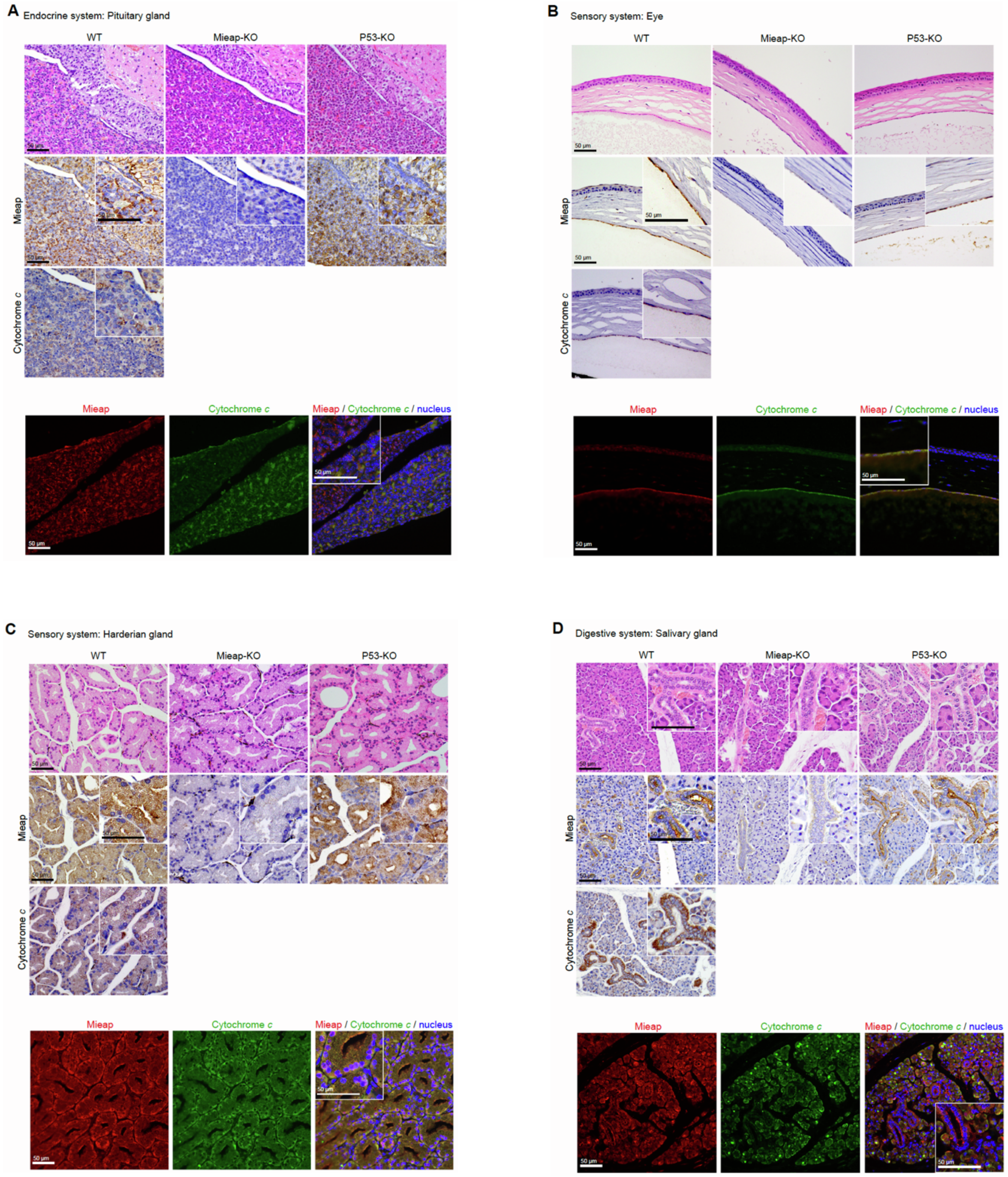

Figure S14. Specific expression of endogenous Mieap protein in pituitary gland, eye, harderian gland, and salivary gland evaluated by histological analysis, related to Figure 8.

(A – D) Hematoxylin and eosin staining (upper panels), diaminobenzidine-based immunohistochemistry (IHC) (middle panels), and immunofluorescence (IF) (lower panels) for Mieap and cytochrome c performed for the WT, Mieap-KO, and p53-KO mice tissues/organs: (A) pituitary gland, (B) eye, (C) harderian gland, and (D) salivary gland. Scale bars, 50 μm.

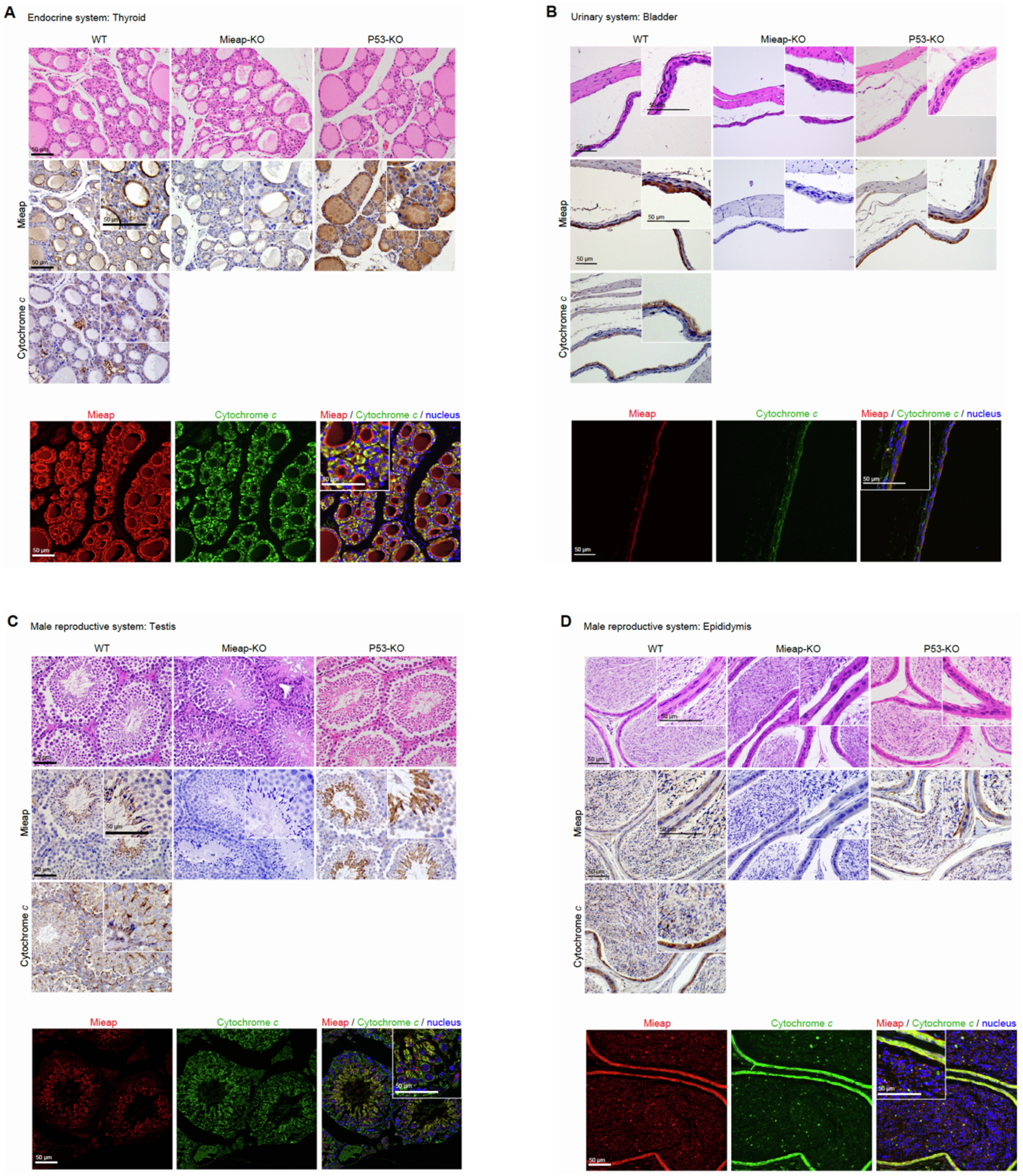

Figure S15. Specific expression of endogenous Mieap protein in thyroid, bladder, testis, and epididymis evaluated by histological analysis, related to Figure 8.

(A – D) Hematoxylin and eosin staining (upper panels), diaminobenzidine-based immunohistochemistry (IHC) (middle panels), and immunofluorescence (IF) (lower panels) for Mieap and cytochrome c performed for the WT, Mieap-KO, and p53-KO mice tissues/organs: (A) thyroid, (B) bladder, (C) testis, and (D) epididymis. Scale bars, 50 μm.

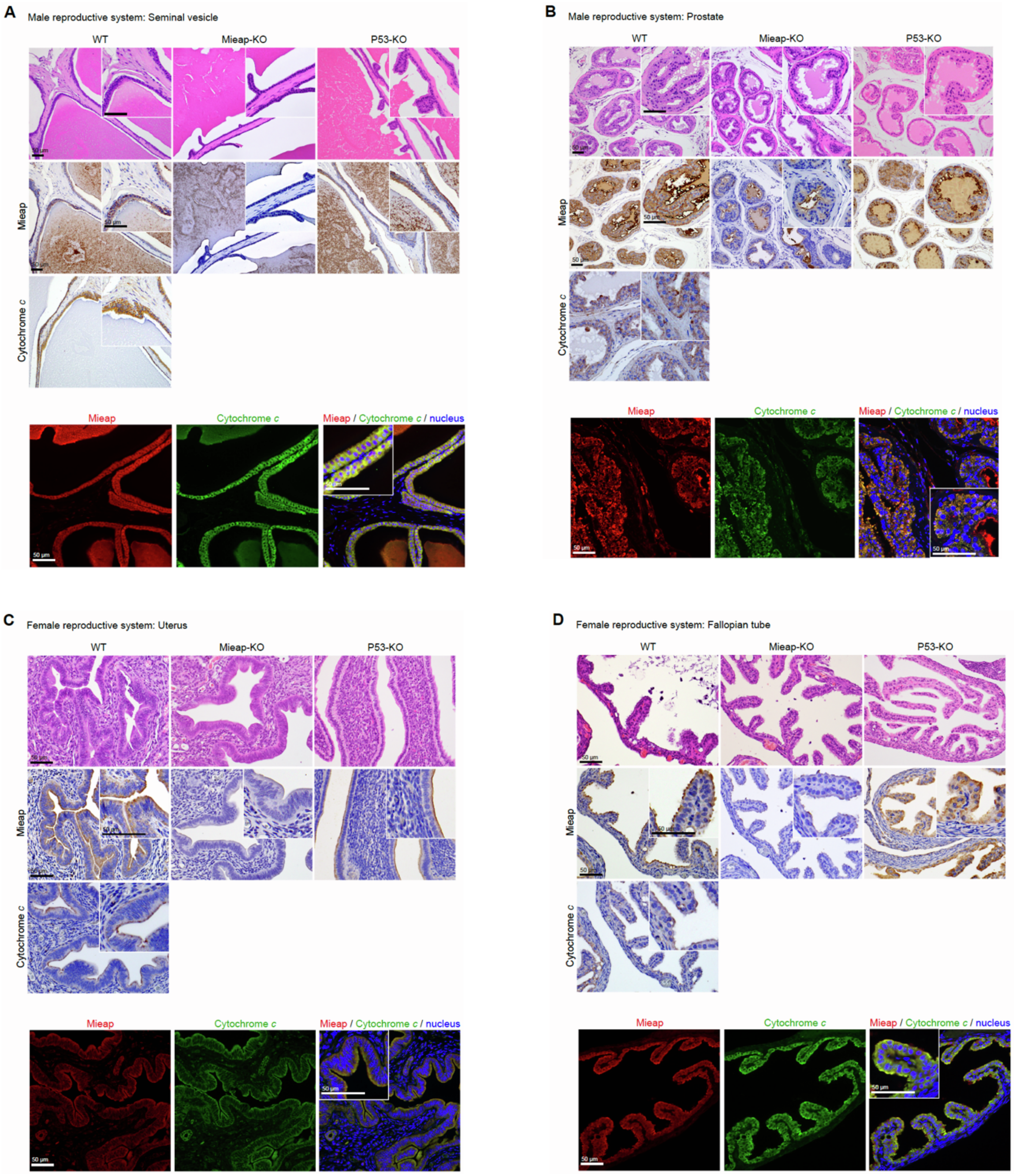

Figure S16. Specific expression of endogenous Mieap protein in seminal vesicle, prostate, uterus, and fallopian tube evaluated by histological analysis, related to Figure 8.

(A – D) Hematoxylin and eosin staining (upper panels), diaminobenzidine-based immunohistochemistry (IHC) (middle panels), and immunofluorescence (IF) (lower panels) for Mieap and cytochrome c performed for the WT, Mieap-KO, and p53-KO mice tissues/organs: (A) seminal vesicle, (B) prostate, (C) uterus, and (D) fallopian tube. Scale bars, 50 μm.
